# A phylogenetically distinctive and extremely heat stable light-driven proton pump from the eubacterium *Rubrobacter xylanophilus* DSM 9941^T^

**DOI:** 10.1038/srep44427

**Published:** 2017-03-14

**Authors:** Kanae Kanehara, Susumu Yoshizawa, Takashi Tsukamoto, Yuki Sudo

**Affiliations:** 1 Division of Pharmaceutical Sciences, Okayama University, Okayama 700-8530, Japan; 2 Atmosphere and Ocean Research Institute, The University of Tokyo, Chiba 277-8564, Japan; 3 Graduate School of Medicine, Dentistry and Pharmaceutical Sciences, Okayama University, Okayama 700-8530, Japan

## Abstract

Rhodopsins are proteins that contain seven transmembrane domains with a chromophore retinal and that function as photoreceptors for light-energy conversion and light-signal transduction in a wide variety of organisms. Here we characterized a phylogenetically distinctive new rhodopsin from the thermophilic eubacterium *Rubrobacter xylanophilus* DSM 9941^**T**^ that was isolated from thermally polluted water. Although *R. xylanophilus* rhodopsin (RxR) is from Actinobacteria, it is located between eukaryotic and archaeal rhodopsins in the phylogenetic tree. *Escherichia coli* cells expressing RxR showed a light-induced decrease in environmental pH and inhibition by a protonophore, indicating that it works as a light-driven outward proton pump. We characterized purified RxR spectroscopically, and showed that it has an absorption maximum at 541 nm and binds nearly 100% all-*trans* retinal. The p*K*_a_ values for the protonated retinal Schiff base and its counterion were estimated to be 10.7 and 1.3, respectively. Time-resolved flash-photolysis experiments revealed the formation of a red-shifted intermediate. Of note, RxR showed an extremely high thermal stability in comparison with other proton pumping rhodopsins such as thermophilic rhodopsin TR (by 16-times) and bacteriorhodopsin from *Halobacterium salinarum* (HsBR, by 4-times).

All organisms have ion transporters in their cell membranes to maintain their cellular activities by regulating an electrochemical potential across the cell membrane. In general, the membrane potential is generated by energy-coupled ion pumps and is transiently dissipated by stimulus-dependent ion channels. Thus ion transporters are essential for all living organisms and are therefore of great interest to many researchers. Since 2005, using photoactivatable ion pumps and channels, the behaviors of living animals have been successfully controlled by light with high temporal and special resolution^1^,^2^. This new technology is called “optogenetics”^3^. In addition to the energetic point of view, ion transporters are one of the most important targets for a wide variety of drugs. Thus ion transporters have become a focus of interest, in part because of their importance to the general understanding both of membrane protein functions and drug development. However, the instability of purified ion transporters, especially in detergent micelles, hampers their characterization.

Rhodopsin is defined as a seven-transmembrane α-helical photoreactive protein having vitamin A aldehyde retinal as a chromophore. The retinal is bound to a specific conserved Lys residue of the apoprotein “opsin” via a protonated Schiff base linkage^4^. Rhodopsins are receiving a lot of attention as a model not only for ion transporters, but also for membrane proteins, because the activity of rhodopsins can be easily judged by their visible color^4^. In addition, since the 21st Century, advances in genomic analysis have revealed that rhodopsins are widespread in the microbial world, including alkalophilic, halophilic and thermophilic organisms^5^. The microbial rhodopsins are categorized as type-1 rhodopsins in comparison with the type-2 animal rhodopsins^5^. Light absorption by type-1 rhodopsins commonly triggers a *trans*-*cis* isomerization of the retinal chromophore, which returns to the original state by sequential photoreaction through a variety of spectroscopically distinctive photointermediates, such as J, K, L, M, N and O^4^. Structural changes of the transmembrane helices and soluble domains are induced during the cyclic photoreaction called the photocycle, which lead to the cognate biological functions^4^.

Among the microbial type-1 rhodopsins, one of the major biological functions is a light-driven electrogenic outward proton pump from the cytoplasmic (CP) side to the extracellular (EC) side, in which the electrochemical potential gradient of proton is utilized for the production of ATP^6^,^7^. Historically, the first type-1 rhodopsin was found in the halophilic archaeon *Halobacterium salinarum* (formerly *halobium*) in 1971 as a light-driven proton pump and it was named *H. salinarum* bacteriorhodopsin (HsBR)^8^. Since then, a large number of proton pumping rhodopsins have been found from various environments worldwide^4^. For instance, proteorhodopsin (PR), *Leptosphaeria* rhodopsin (LR), xanthorhodopsin (XR) and thermophilic rhodopsin (TR) were found in the eubacterium marine bacterioplankton in 2000^9^, in the eukaryotic fungus *Leptosphaeria maculans* in 2005^10^, in the halophilic eubacterium *Salinibacter ruber* in 2005^11^, and in the thermophilic eubacterium *Thermus thermophilus* in 2013^12^, respectively. Thus, the wide distribution of proton pumping rhodopsins indicates that a previously unsuspected mode of microbially mediated light-driven ATP production commonly occurs on earth.

On the basis of that background, we report here a new eubacterial proton pumping rhodopsin from the gram-positive thermophilic eubacterium *Rubrobacter xylanophilus* DSM 9941^**T**^, which is distinguishable from eukaryotic and archaeal proton pumps (Fig. 1a). *R. xylanophilus* was found in a filter of thermally polluted water in a carpet factory in England, and its optimum pH and temperature were reported to be pH 7.5–8.0 and 60 °C, respectively^13^. In this study, the codon-optimized gene for the new rhodopsin, named *R. xylanophilus* rhodopsin (RxR), was chemically synthesized and then was heterologously expressed in *Escherichia coli* as a recombinant protein. We characterized the purified RxR spectroscopically and its properties were compared with those of other microbial proton pumping rhodopsins. Of note, thermal denaturation experiments revealed that RxR maintained its visible absorption even at 85 °C for more than 10 hours, indicating that RxR is the most thermally stable microbial rhodopsin discovered so far.

## Results and Discussion

### Functional characterization of RxR

**Figure 1:**
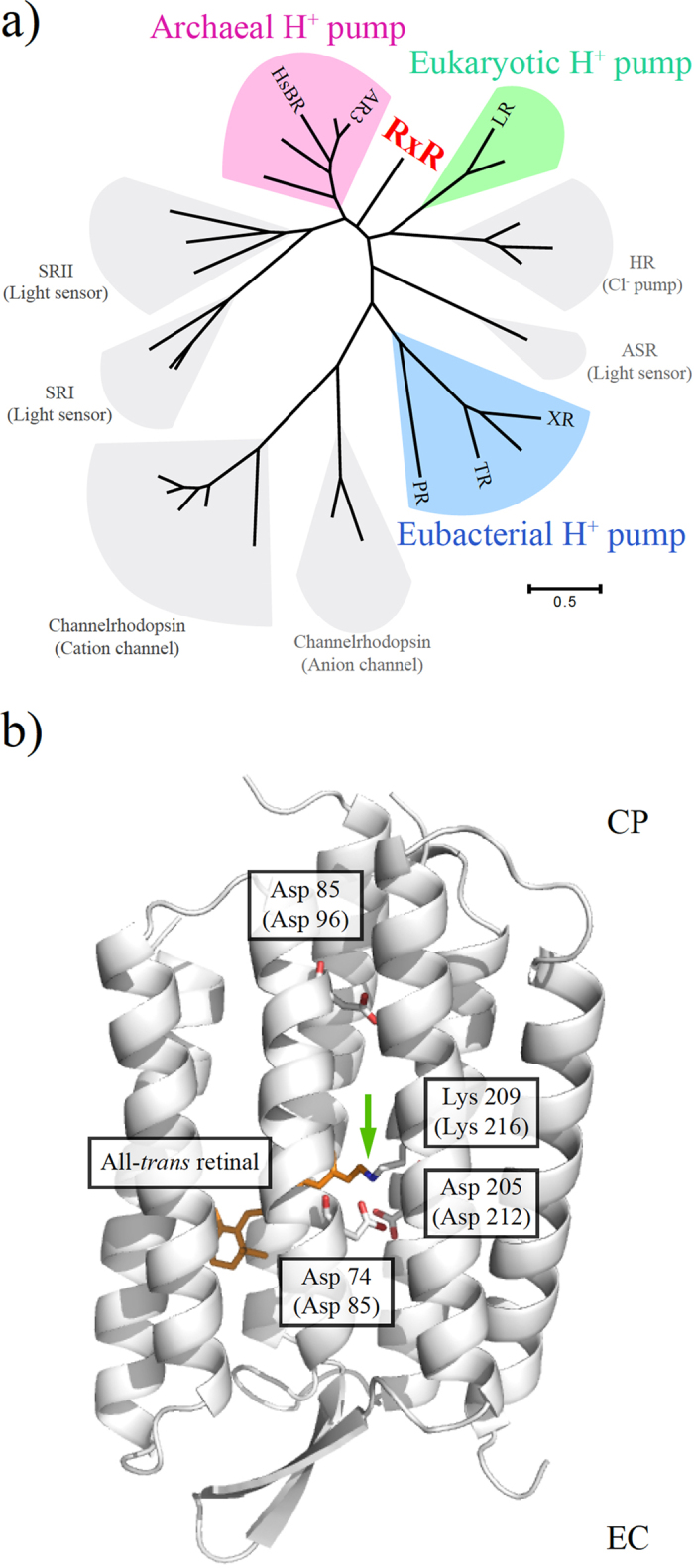
Phylogenetic and structural introduction of microbial rhodopsins. (**a**) Unrooted maximum likelihood tree of microbial rhodopsin amino acid sequences. Amino acid sequences of microbial rhodopsins including RxR were aligned using MUSCLE^34^, and evolutionary distances were estimated using the JTT matrix-based method^35^. The Maximum Likelihood tree was constructed using bootstrap values based on 1000 replications; evolutionary analyses were conducted in MEGA6^36^. All rhodopsin amino acid sequence data used in this study were obtained from the public database (http://www.ncbi.nlm.nih.gov/). They are categorized into Archaeal H^+^ pumps, including HsBR and AR3, SRI (sensory rhodopsin I), SRII (sensory rhodopsin II), Channelrhodopsins, including cation- and anion-channelrhodopsins, Eubacterial H^+^ pumps, including PR, XR and TR, ASR (Anabaena sensory rhodopsin), HR (halorhodopsin), Eukaryotic H^+^ pumps, including LR, and RxR. These microbial rhodopsins are distributed into all domains of life (eubacteria, eukarya and archaea). Among them, the proton pumping rhodopsins are colored pink, blue and green, respectively. The scale bar represents the number of substitutions per site. (**b**) Crystal structure of an outward proton pump HsBR (PDB code: 1C3W)^37^. The all-*trans* retinal chromophore binds to the apoprotein opsin via a protonated Schiff base linkage with a conserved lysine residue. All charged residues critical for the proton pump function in HsBR, Asp85, Asp96, Asp212 and Lys216, are also conserved in RxR as Asp74, Asp85, Asp205 and Lys209. The green arrow indicates the Schiff base linkage. The membrane normal is roughly in the vertical plane, and the top and bottom regions correspond to the cytoplasmic (CP) and extracellular (EC) sides, respectively.

As described above, recent extensive genomic investigations have revealed the wide distribution and rich diversity of microbial rhodopsins in nature^5^. Figure 1a provides a brief phylogenetic introduction to microbial rhodopsins. To compare the phylogenetic relationships and distances between RxR and the other known microbial rhodopsins with different, individual functions, we selected 29 amino acid sequences of the rhodopsins including 5 archaeal proton pumps, 4 bacterial ones, and 2 eukaryotic ones from the public database (http://www.ncbi.nlm.nih.gov/). As seen, the archaeal proton pumps, including HsBR and archaerhodopsin-3 (AR3) (colored pink), eukaryotic ones, including LR (colored green) and eubacterial ones, including PR, XR and TR (colored blue), form distinct phylogenetic clades. In addition to the proton pumping rhodopsins, there are functionally different rhodopsins that serve as chloride ion pumps (halorhodopsin, HR), light sensors (sensory rhodopsins I and II, SRI and SRII, and Anabaena sensory rhodopsin, ASR) and ion channels (cation- and anion-channelrhodopsins). A putative rhodopsin protein RxR was discovered in the thermophilic eubacterium *R. xylanophilus* in 2014 (GenBank ID: ABG04982). Although RxR is produced in a eubacterium, its amino acid sequence is most closely related to the archaeal proton pumps (44% identity, 71% similarity in comparison with HsBR and 49% identity, 81% similarity in comparison with AR3) and eukaryotic proton pumps (37% identity, 68% similarity in comparison with LR), but not to eubacterial proton pumps (21% identity, 59% similarity in comparison with PR, 23% identity, 63% similarity in comparison with XR, and 25% identity, 59% similarity in comparison with TR). Thus, in the phylogenetic tree, RxR is distinguished from archaeal and eukaryotic proton pumps (Fig. 1a). In the previous papers^14^,^15^, we used only prokaryotic rhodopsins to make a phylogenetic tree. On the other hand, we included eukaryotic rhodopsins such as LR, *Neurospora* rhodopsin (NR) and channelrhodopsins in this study to compare the location of RxR precisely. Therefore, the phylogenetic tree seems to be mildly different from those of published so far^14^,^15^.

Regarding the amino acid sequence, RxR contains characteristic charged amino acids known to be important for proton pumps, Arg71 (Arg82 in HsBR), Asp74 (Asp85 in HsBR), Thr78 (Thr89 in HsBR), Asp85 (Asp96 in HsBR), Glu187 (Glu194 in HsBR), Glu197 (Glu204 in HsBR), Asp205 (Asp212 in HsBR) and Lys209 (Lys216 in HsBR) as well as other proton pumping rhodopsins (Fig. 1b and Supplemental Figure S1). In HsBR, Lys216 composes the site of the protonated Schiff base linkage with Asp85, Asp212 and water molecules^6^. During the photoreaction, Asp85 and Asp96 work as a proton acceptor and a donor for the protonated and the deprotonated Schiff base, respectively^16^. Thus, based on those conserved residues, RxR is expected to act as a light-driven outward proton pump.

**Figure 2:**
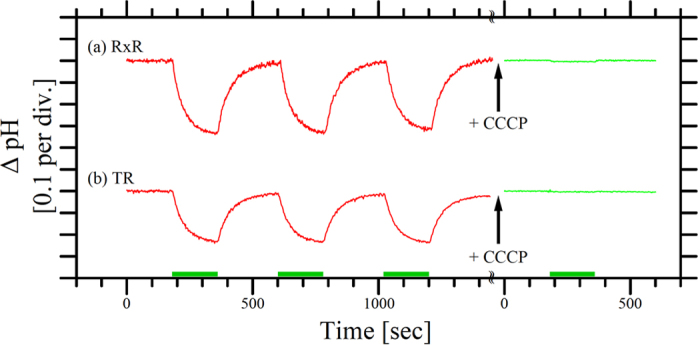
Proton pumping activity of RxR. Light-induced pH changes of *E. coli* BL21(DE3) cells expressing RxR (**a**) and TR (**b**). The initial pH values were 6.2 for RxR and 6.3 for TR. The cell suspension was illuminated with green light (540 nm, ca. 14–16 mW/cm^2^, green bars) in a solution containing 100 mM NaCl and pH changes were observed in the absence (red lines) and presence (green lines) of the protonophore CCCP (final concentration 40 μM). The temperature was maintained at 25 °C using a thermostat.

To confirm whether RxR encodes a functional protein, we prepared a codon-optimized plasmid DNA encoding RxR to express it in *E. coli* cells as a recombinant protein. Detailed sequence was shown in Supplemental Figure S2. Figure 2a shows the light-induced pH change of a suspension of *E. coli* cells expressing RxR, which was successfully confirmed by the dark pink color of the cells. The similar experiments were performed for TR as a reference (Fig. 2b). As seen, a light-induced decrease in pH (acidification of the medium) was observed (red line) both for RxR (a) and TR (b), implying proton movement from the CP to the EC side upon illumination. The altered pH gradually returned to the original level after turning off the light. To check the light-induced protein unfolding (denaturation), the samples were repeatedly irradiated by light. As seen, the second and third signals were almost comparable to the first signal, indicating that the samples were not denatured upon light irradiation. On the other hand, the pH change was strongly impaired in the presence of the protonophore carbonyl cyanide m-chlorophenylhydrazone CCCP (green line). Thus, these data indicate that RxR is a light-driven outward electrogenic proton pump. We assumed that the proton is translocated through carboxylates Asp74 and Asp85 in RxR as occurs in HsBR. To examine whether RxR is expressed in the native *R. xylanophilus* DSM 9941^T^ strain, we obtained it from the national culture collection. However, the native cell has not been grown in the standard culture medium for eubacteria. We are optimizing the contents of the culture medium.

### Spectroscopic characterization of RxR

**Figure 3:**
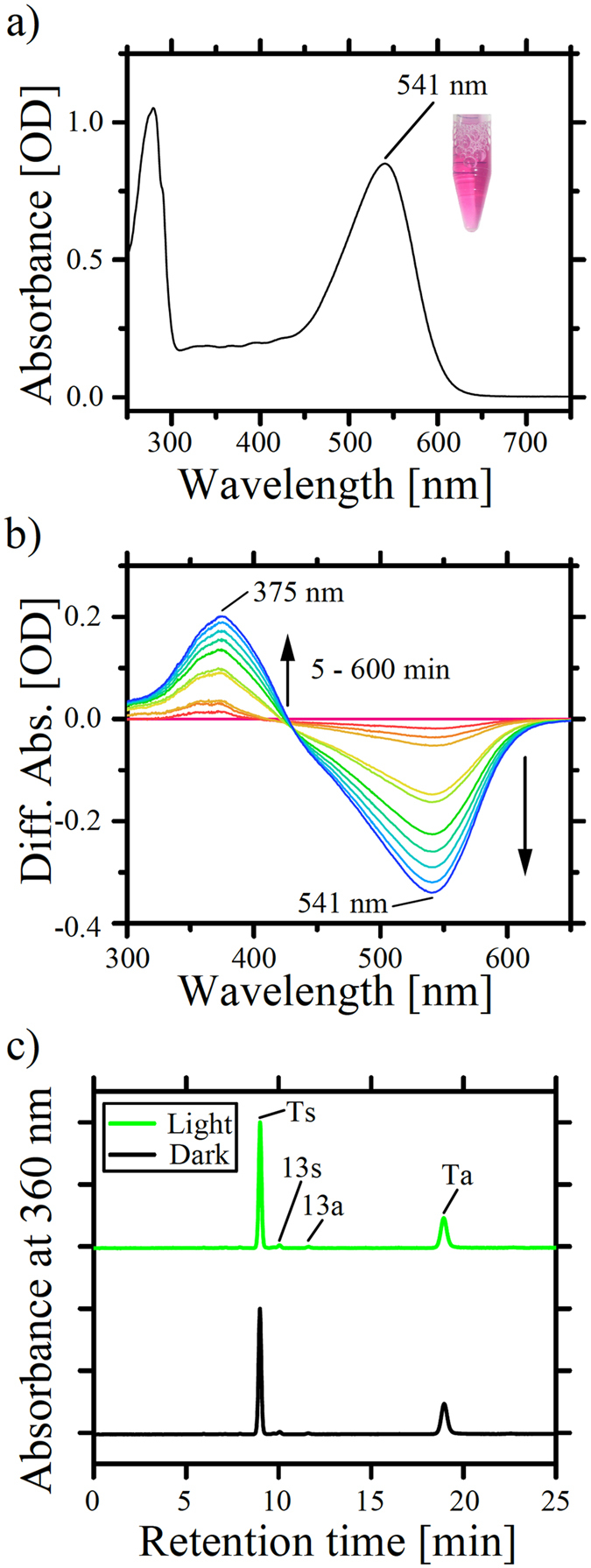
Absorption spectrum of RxR and its retinal configuration. (**a**) UV-Visible absorption spectrum of purified RxR in a buffer containing 50 mM Tris-HCl (pH 7.0), 1 M NaCl and 0.05% DDM. (**b**) Difference UV-Visible spectra of RxR at varying times after the addition of hydroxylamine (final concentration, 100 mM). (**c**) HPLC patterns of RxR retinal oxime with (light) and without (dark) green light irradiation (540 nm) for 3 min. The symbols, Ts, Ta, 13 s and 13a, represent all-*trans*-15-*syn*, all-*trans*-15-*anti*, 13-*cis*-15-*syn*, and 13-*cis*-15-*anti* retinal oxime, respectively. The molar compositions of each retinal isomer were calculated from the areas of the peaks in the HPLC patterns.

To investigate the photochemical properties of RxR, we carried out spectroscopic measurements of the purified RxR protein. The UV-Visible absorption spectrum of RxR showed that its absorption maximum (λ_max_) was located at 541 nm (Fig. 3a), which is almost identical to LR (542 nm)^10^ and is significantly different from HsBR (570 nm)^6^ and TR (530 nm)^12^. We then estimated the molar extinction coefficient value for RxR at λ_max_ (541 nm) by utilizing the reaction of RxR with hydroxylamine (Fig. 3b). It is known that the chromophore retinal is released from the rhodopsin as a retinal oxime by the reaction between the retinylidene Schiff base and hydroxylamine, resulting in the spectral blue-shift from the visible region to around 370 nm^17^. As expected, decreases in absorption at 541 nm are observed for the purified RxR with concomitant increases in the absorption at 375 nm (Fig. 3b). From the ratio between these absorption changes and the known molar extinction coefficient value for the retinal oxime (33,600 M^−1^ cm^−1^)^17^, we calculated the molar extinction coefficient value for RxR at λ_max_ (541 nm) as 54,000 M^−1^ cm^−1^. To investigate the retinal configurations, an experiment using High-performance liquid chromatography (HPLC) was then carried out. Figure 3c shows the HPLC chromatograms of retinal isomers extracted from RxR under dark (black line) and light (green line) conditions. The assignment of each peak was performed by comparing it with the HPLC pattern from retinal oximes of authentic all-*trans*- and 13-*cis*-retinals as reported previously^18^. Two peaks at retention times of around 9 min and 19 min were mainly observed and were assigned as the all-*trans*-15-*syn* configuration (denoted as Ts) and the all-*trans*-15-*anti* configuration (denoted as Ta), respectively. Additionally, two small peaks were also observed at retention times of around 10 min and 11.6 min, which were assigned as the 13-*cis*-15-*syn* configuration (denoted as 13 s) and the 13-*cis*-15-*anti* configuration (denoted as 13a), respectively. Finally, the retinal composition ratio (%) in the dark and light were estimated from the area of each peak to be all-*trans*:13-*cis* = 98.3:1.7 and 97.8:2.2, respectively. It turns out that RxR predominantly possesses the all-*trans* retinal isomer under both dark- and light-adapted conditions. It is known that light-driven archaeal ion pumps, such as BR and HR, possess both all-*trans* (30–50%) and 13-*cis* retinal (50–70%) in the dark^19^,^20^, whereas the other eubacterial and eukaryotic ion pumps, such as PR, TR, XR and LR, possess all-*trans* retinal predominantly^10^,^12^,^21^,^22^. Thus, the retinal composition of RxR reflects the retinal composition of eubacterial and eukaryotic rhodopsins.

**Figure 4:**
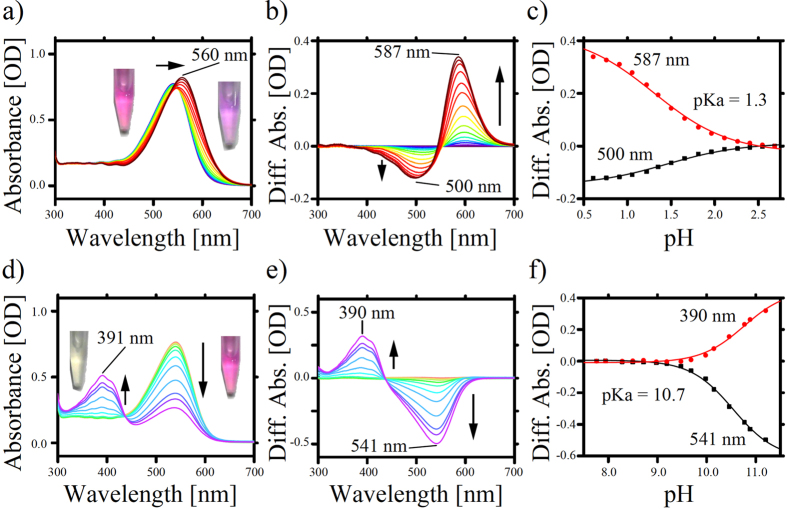
pH-induced absorption changes in RxR. The sample was suspended in a buffer consisting of a mixture of 6 buffering agents (0.89 mM citric acid, 0.89 mM MES, 1.1 mM TES, 0.78 mM TAPS, 1.1 mM CHES and 0.33 mM CAPS) with 0.05% DDM. (**a**) Absorption spectra at acidic pH 0.609–2.686; the pH was adjusted to the desired value by adding concentrated H_2_SO_4_. (**b**) Difference spectra at acidic pH 0.609–2.686; the spectrum at pH 2.686 was subtracted from each spectrum and is described as a baseline. (**c**) Estimation of the p*K*_a_ of the counterion; absorption changes at 587 nm (red circles) and at 500 nm (black squares) were plotted against pH values; data were analyzed using the Henderson-Hasselbalch equation with a single p*K*_a_ value (solid lines). (**d**) Absorption spectra obtained at alkaline pH 7.761–11.203; the pH was adjusted to the desired value by adding concentrated KOH. (**e**) Difference spectra at alkaline pH 7.761–11.203; the spectrum at pH 7.761 was subtracted from each spectrum and is described as a baseline. (**f**) Estimation of the p*K*_a_ of the protonated Schiff base. Absorption changes at 390 nm (red circles) and at 541 nm (black squares) were plotted against pH values; data were analyzed using the Henderson-Hasselbalch equation with a single p*K*_a_ value (solid lines). The p*K*_a_ values were estimated as 1.3 ± 0.17 and 10.7 ± 0.03 for the counterion (presumably Asp74) and the Schiff base (Lys209), respectively. All titration experiments were performed at room temperature (approx. 25 °C).

It is well-known that protons (H^+^) are rapidly and indirectly transferred through functional groups inside the proteins via the Grotthuss mechanism^6^, whereas direct translocation is needed for other ions. Therefore, charged residues, such as Asp, Glu and Lys, play an essential role in the proton pumping rhodopsins^4^. Spectroscopic pH titration experiments were then performed to estimate the p*K*_a_ values of the charged residues in RxR. As shown in Fig. 4a, a spectral red-shift from 541 nm to 560 nm was observed under acidification conditions. In rhodopsins, the protonation of the counterion of the protonated Schiff base leads to a decrease in the energy gap between the electronic ground and excited states, resulting in a spectral red-shift^23^. Therefore, the acid-induced spectral shift in RxR represents the protonation of the counterion. From analogy with other microbial rhodopsins^4^, the most favorable candidate for the counterion is Asp74 in RxR (Asp85 in HsBR). The difference absorption spectra showed increases in absorbance at 587 nm and concomitant decreases in absorbance at 500 nm with an isosbestic point at around 545 nm, indicating the presence of an equilibrium between the protonated and deprotonated forms of the counterion in RxR (Fig. 4b). To estimate its p*K*_a_ value, the absorption changes at 587 nm (red circles) and 500 nm (black squares) were plotted against the environmental pH (Fig. 4c). Both data were fitted well by the Henderson-Hasselbalch equation with a single p*K*_a_ and the p*K*_a_ value for the counterion (presumably Asp74) was estimated to be 1.3 ± 0.17. The value for RxR is a few units lower than those of other rhodopsins.

Similarly, titration experiments were performed in an alkaline pH. As shown in Fig. 4d, a spectral blue-shift from 541 nm to 391 nm was observed under alkaline conditions. It has been reported that deprotonation of the Schiff base nitrogen leads to an increase in the energy gap between the electronic ground and excited states, resulting in a spectral blue-shift^23^, and the absorption maximum of the retinal chromophore with the deprotonated Schiff base nitrogen appeared at around 390 nm^24^. Thus, the spectral shift can be assigned to be the deprotonation of the Schiff base nitrogen of Lys209 in RxR (Lys216 in HsBR). The difference absorption spectra showed increases in absorbance at 390 nm and concomitant decreases in absorbance at 541 nm with an isosbestic point at around 420 nm, indicating the presence of an equilibrium between the protonated and deprotonated forms of the Lys209 in RxR (Fig. 4e). From the ratio of the areas of the two peaks, it is likely that the RxR having a deprotonated Schiff base has roughly a two-fold larger extinct coefficient than the RxR having a protonated one. To estimate its p*K*_a_ value, the absorption changes at 390 nm (red circles) and 541 nm (black squares) were plotted against the environmental pH (Fig. 4f). Both data were fitted well by the Henderson-Hasselbalch equation with a single p*K*_a_ and the p*K*_a_ value for Lys209 in RxR was estimated to be 10.7 ± 0.03, which is a few units lower than those of other rhodopsins. From these results, it is assumed that RxR has a salt bridge between the deprotonated counterion (p*K*_a_ = 1.3) and the protonated Schiff base (p*K*_a_ = 10.7) in native cells, because *R. xylanophilus* lives at a weak alkaline pH (7.5–8.0) as described above^13^.

**Figure 5:**
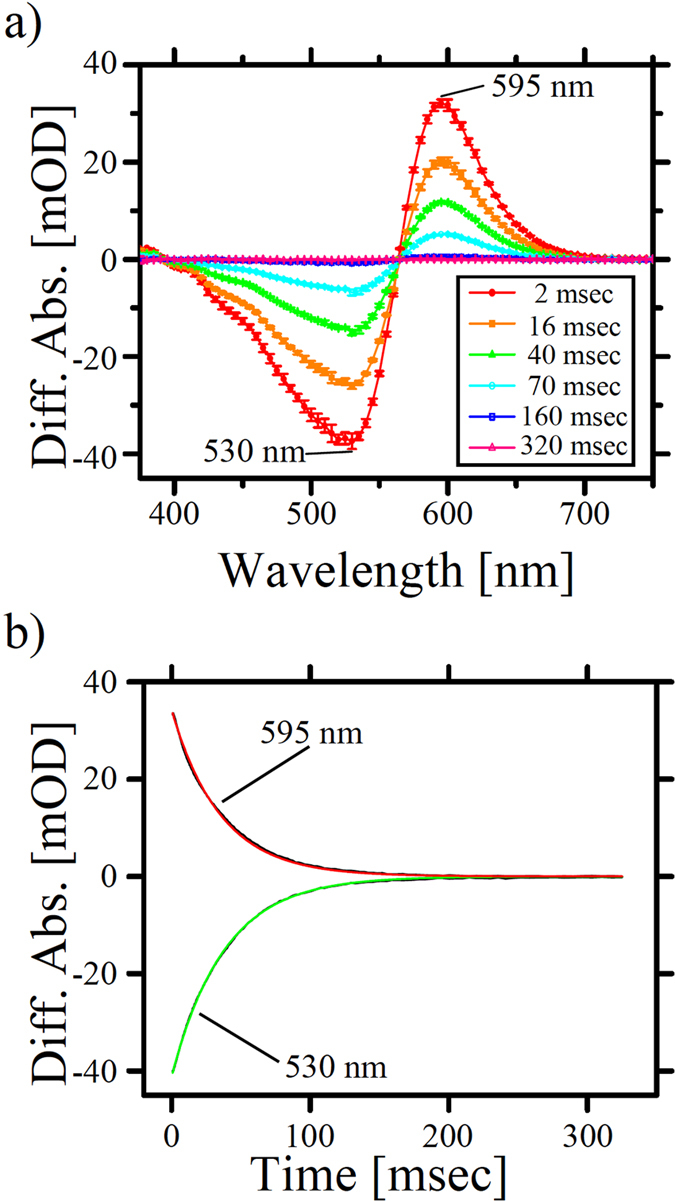
Photoreaction kinetics of RxR. (**a**) Flash-induced difference absorption spectra of RxR at 60 °C over a spectral range of 375 to 750 nm and a time range of 2 ms to 320 ms. Depletion of the original state (530 nm) and a simultaneous increase in the O-like red-shifted (595 nm) intermediate were observed with an isosbestic point at around 565 nm. Error bars indicate the standard deviations of three independent experiments (n = 3). (**b**) Flash-induced kinetic data of RxR at 530 nm (green) and 595 nm (red) representing recovery of the original state and the O-decay, respectively.

We then performed transient time-resolved flash-photolysis experiments to investigate the photoreaction kinetics of RxR from the millisecond to second time domains, where blue-shifted M-like and red-shifted O-like intermediates were mainly observed in microbial rhodopsins^4^. In these experiments, the pH was adjusted to 7.4, where the Schiff base nitrogen and its counterion are protonated and deprotonated, respectively, and the temperature was kept at a physiological level (60 °C) using a thermostat. Figure 5a shows the flash-induced difference spectra of the purified RxR over the spectral range of 375–750 nm. The depletion and recovery at around 530 nm are attributable to the absorption changes of the original state because the peak of the signals is similar to the absorption maximum of RxR (541 nm) as shown in Fig. 3a. In addition, the formation and decay observed at around 595 nm were attributable to the O-like (RxR_O_) intermediate (Fig. 5a), according to their absorption maxima and the sequence of intermediates observed in the other proton pumping rhodopsins^6^,^9^,^10^,^12^. The spectral shoulder was observed at around 430 nm. Although the origin is still unclear, we speculate that it may be a contribution of some intermediate(s) with shorter absorption wavelength.

Figure 5b shows the time courses of the absorbance change at selected wavelengths; 530 nm for original RxR and 595 nm for RxR_O_ (black lines). All curves were fitted well to a single exponential equation (green and red lines, respectively in Fig. 5b) and the recovery rate constant of the photocycle was estimated as 2.8 × 10^−2^ msec^−1^, which is similar to that of other proton pumping rhodopsins and is much smaller than that of sensory rhodopsins^25^,^26^. The fast photocycle makes RxR an efficient proton pump because one proton is transferred from the CP to the EC side during a single photocycle. On the basis of these results, we assumed the photoreaction and proton translocation pathway of RxR from the analogy with other proton pumping rhodopsins. The all-*trans* retinal chromophore is isomerized to 13-*cis* upon formation of the early photointermediate(s). A proton of the Schiff base nitrogen is transferred to the counterion (presumably Asp74) upon formation of the M-like intermediate and the deprotonated Schiff base is reprotonated by the proton donor Asp85 (Asp96 in HsBR) during the decay of the M-like intermediate, where relatively large conformational changes of the protein moiety were induced. Upon formation of the O-like intermediate, the isomerized retinal chromophore returns from 13-*cis* to all-*trans.* Finally, the photocycle is completed with the O-decay. The time resolution of our strobe flash-photolysis system used here is about 1 ms, which is not enough to detect early photointermediate(s). We would like to investigate both the origin of the spectral shoulder at around 430 nm (see Fig. 5a) and the sequence of proton movement in near future by using laser-flash photolysis and a proton sensitive dye such as pyranine.

### Extremely high thermal stability of RxR

As described above, RxR was obtained from a thermophilic organism^13^, and therefore it is expected that RxR is resistant to thermal irradiation. We used TR and HsBR as references because the thermal stabilities of them are much larger than the other rhodopsins^12^,^15^,^27^. Here TR was purified by the same methods as described previously^15^. Of note, before the measurement, the purified RxR, TR, and the detergent-solubilized HsBR were dialyzed against the same buffer containing 50 mM Tris-HCl (pH 7.0), 1 M NaCl and 0.05% n-dodecyl-β-D-maltoside (DDM) for more than 2 weeks to properly control the concentration of DDM, because it is known that the concentration of DDM affects the stability of proteins^28^. In the previous studies, based on two criteria, (i) disappearance of the isothermal titration calorimetry signal caused by dilution heat of the detergent DDM between analytes and ligands^29^ and (ii) the intensity of ^1^H-NMR signals from the detergent DDM^30^, we have found that it takes at least 1 week until the detergent concentration within the sample solution reaches equilibrium with that in the external dialysis buffer solution. Although, in this study, we did not check the level of residual DDM concentration, it should be close to 0.05% because the samples were dialyzed against the same buffer for more than 2 weeks.

**Figure 6:**
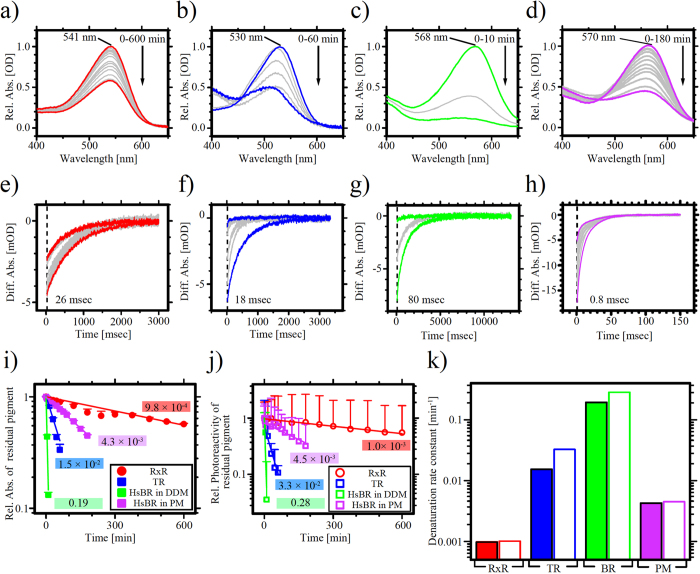
Thermal stability of RxR. (**a**–**d**) Time-dependent decreases in the absorbance at 541 nm for RxR (**a**), at 530 nm for TR (**b**), at 568 nm for HsBR in DDM (**c**), and at 570 nm for HsBR in purple membrane (PM) (**d**). The detergent-solubillized samples were suspended in the same buffer containing 50 mM Tris-HCl (pH 7.0), 1 M NaCl and 0.05% DDM and the PM was suspended in a buffer containing 50 mM Tris-HCl (pH. 7.0) and 1 M NaCl without DDM. The protein concentration of each sample was adjusted to 2 μM. The temperature was kept at 85 °C using a thermostat. (**e**–**h**) Residual photoreactivity of RxR at 530 nm (**e**), TR at 530 nm (**f**), HsBR in DDM at 560 nm (**g**) and HsBR in PM at 560 nm (**h**). (**i**) Denaturation kinetics at 85 °C obtained from time-dependent decreases in visible absorbance (panels a–d) of RxR (red circles), TR (blue squares), HsBR in DDM (green squares) and HsBR in PM (purple squares). (**j**) Denaturation kinetics at 85 °C obtained from time-dependent decreases in the photoreactivity (panels e–h). For panels (i) and (j), error bars indicate the standard deviations of three independent experiments (n = 3). The solid lines represent fitting curves of a single exponential function. (**k**) Comparison of the denaturation rate constants of RxR (red blocks), TR (blue blocks), HsBR in DDM (BR, green blocks), and HsBR in PM (purple blocks). Filled and open blocks were obtained from the data of panels (i) and (j), respectively.

We then investigated the thermal stability of RxR and compared it with TR and HsBR. Figure 6 shows the time-dependent thermal denaturation of RxR (a), TR (b), HsBR in DDM (c), and HsBR in purple membrane (PM) (d) at 85 °C (358 K) monitored by UV-Visible spectroscopy over a spectral range of 400 to 650 nm. TR lost its absorbance within 60 min with a slight (a few nanometers) spectral blue-shift due to the trimer-monomer transition as reported previously^31^. HsBR in DDM lost its absorbance within 10 min whereas HsBR in PM held its absorbance for 180 min. On the other hand, RxR held its absorbance at 541 nm during the 600 min incubation time and more than 50% of the protein retained the color without a significant spectral shift. The residual absorption upon thermal irradiation was then plotted against the incubation time (Fig. 6i). The denaturation rate (time) constants were estimated using a single exponential function and resulted in 9.8 × 10^−4^ min^−1^ (τ_1/2_ = 12 hr) for RxR, 1.5 × 10^−2^ min^−1^ (τ_1/2_ = 0.75 hr) for TR, 0.19 min^−1^ (τ_1/2_ = 6.0 × 10^−2^ hr) for HsBR in DDM, and 4.3 × 10^−3^ min^−1^ (τ_1/2_ = 2.7 hr) for HsBR in PM (Fig. 6k), indicating that the thermal stability of RxR is 16-times larger than that of TR and 4-times larger than that of HsBR in PM. Furthermore, we examined the photoreactivity of these rhodopsins after incubation at 85 °C by flash-photolysis measurements. We confirmed that all the rhodopsins used here showed the photoreactivity even after the incubation at 85 °C. To examine whether the photocycle of RxR after heat irradiation is normal, we obtained flash-induced kinetic data of RxR before and after heat irradiation as shown in Supplementary Figure S3. As a result, the kinetic traces for RxR after heat irradiation are almost identical to those before heat irradiation, indicating that non-denaturing RxR after heat irradiation shows normal photoreactivity. In Fig. 6j, we plotted the maximum difference absorbance at 26 ms for RxR, at 18 ms for TR, at 80 ms for HsBR in DDM, and at 0.8 ms for HsBR in PM against incubation time and analyzed by a single exponential decay function (solid lines). From the analysis, we estimated denaturation rates of each molecule as 1.0 × 10^−3^ min^−1^ (τ_1/2_ = 11 hr) for RxR, 3.0 × 10^−2^ min^−1^ (τ_1/2_ = 0.35 hr) for TR, 0.28 min^−1^ (τ_1/2_ = 4.1 × 10^−2^ hr) for HsBR in DDM, and 4.5 × 10^−3^ min^−1^ (τ_1/2_ = 2.6 hr) for HsBR in PM (Fig. 6k). The denaturation rate constants estimated by flash-photolysis were comparable to those estimated by the static absorption spectroscopy shown in Fig. 6i, suggesting that we successfully characterized the biological activities of RxR, TR and BR by two different indexes (visible absorption and photoreactivity). These results indicate that RxR shows much a higher thermal stability than TR (by 16-times), HsBR in DDM (BR) (by 200-times) and HsBR in PM (BR) (by 4-times). Thus, we demonstrated here that RxR is the most stable rhodopsin reported so far. The high stability of RxR will help to characterize the ion transport mechanism in detail and allows to develop tools for the artificial photoreactive system including optogenetics technology. In this study, we characterized RxR using spectroscopic techniques and the properties were compared with those of other high stable rhodopsins (TR and HsBR) under the same experimental conditions including instruments, procedures, media and temperature. In future, to determine the activation energy (*E*_a_) for the denaturation of RxR, we would like to perform further experiments under varying temperatures.

In conclusion, we characterized the phylogenetically distinctive proton pump RxR and found that it shows an extremely high thermal stability. We would like to elucidate the thermal stabilization mechanism of RxR in the near future using research techniques such as molecular biological and structural biological methods. We then plan to produce a hyper-stable rhodopsin and utilize it not only for understanding the biological functions of rhodopsins, but also for optogenetics.

## Methods

### Sample preparation

The *E. coli* strains, DH5α and BL21(DE3), were used as hosts for DNA manipulation and for protein expression, respectively. A gene for RxR was chemically synthesized by Eurofins Genomics (Tokyo, Japan) with NdeI and XhoI restriction enzyme sites at each terminus, where the codon was optimized for *E. coli*. During the optimization, some nucleotides (163 out of 717, 22.7%) were substituted but without any changes in the amino acid sequence (Supplemental Figure S2). The DNA fragment was inserted into the pET21c(+) vector using the NdeI and XhoI restriction enzyme sites and the plasmid was analyzed using an automated sequencer to confirm the expected nucleotide sequence. Consequently, the plasmid encodes hexahistidines at the C-terminus to allow purification of the expressed protein. *E. coli* cells harboring the plasmid were grown at 37 °C in LB medium supplemented with ampicillin (final concentration, 50 μg/mL). The expression of RxR was induced at an optical density at 660 nm (OD_660_) of 1.4–1.6 with 1 mM isopropyl β-D-1-thiogalactopyranoside (IPTG) and 10 μM all-*trans* retinal. The cells were disrupted by sonication (UD-211, TOMY Seiko Co., Ltd., Tokyo, Japan) for 30 min on ice-cold water in a buffer containing 50 mM Tris-HCl (pH 7.0). The crude membrane fraction was collected by ultracentrifugation (40,000 rpm for 60 min, CP 56G ultracentrifuge with P50A2 angle rotor, Hitachi Koki Co., Ltd., Tokyo, Japan) and solubilized with 1.5 w/v % n-dodecyl-β-D-maltoside (DDM, Dojindo, Japan). The solubilized-fraction was purified by Ni^2+^ affinity column chromatography with a linear gradient of imidazole as described previously^32^. The purified protein was concentrated by centrifugation using an Amicon Ultra filter (30,000 MW cutoff; Merk Millipore, Bedford, MA, USA). The sample media was then replaced by the appropriate buffer solution by ultrafiltration for more than 5-times.

*Halobacterium salinarum* ET1001 strain (a kind gift from Dr. Takashi Kikukawa) was grown by the same method reported elsewhere^33^. The cells aerobically grown on agar were transferred to 5 mL liquid culture medium and shaken at 37 °C for about 1 week in the dark. Then, the grown cells were transferred to 500 mL culture medium and shaken under the same condition. Finally, the grown cells were transferred to 2 L culture medium and aerobically shaken at 37 °C for about 2 weeks in the dark and then anaerobically shaken at 37 °C for about 1 week in the light. After harvesting the cells by centrifugation, the cells suspended in 50 mM 2-[4-(2-Hydroxymethyl)-1-piperazinyl] ethanesulfonic acid (HEPES)-NaOH buffer (pH 7.0) containing 3 M NaCl were disrupted by the repeated freeze-thaw cycle. To reduce the viscosity, the cell suspension was stirred at 4 °C overnight in the presence of DNase (ca. 1 mg for 10 g wet cells) and 5 mM MgCl_2_. The purple membrane (PM) was collected by the ultracentrifugation (40,000 rpm for 60 min) and then sufficiently washed by 50 mM HEPES-NaOH buffer (pH 7.0) containing 100 mM NaCl. During the washing process, precipitated impurities were carefully removed. To remove residual carotenoids, the purple membrane was washed by ultrapure water sufficiently. The buffer was exchanged by 3-times by using the ultracentrifuge. The PM was solubilized in the buffer containing 10 mM Tris-HCl (pH 7.0) and 50 mM n-octyl-β-D-glucoside (OG, Calbiochem, Germany) at 37 °C for 2 hr. Then the HsBR was ultracentrifuged (30,000 rpm for 1 hr at 4 °C, CP 56G ultracentrifuge with P50A2 angle rotor, Hitachi Koki Co., Ltd., Tokyo, Japan). After the concentration of the supernatant, it washed by the buffer containing 50 mM Tris-HCl, 1 M NaCl, and 0.05% DDM. Then it was dialyzed for more than 2 weeks at 4 °C in the same buffer containing 50 mM Tris-HCl, 1 M NaCl, and 0.05% DDM.

### Ion transport measurements

*E. coli* cells were grown in the 2 × YT medium and the expression of RxR was induced at an OD_660_ of 0.4–0.6 with 0.1 mM IPTG and 10 μM all-*trans* retinal. The RxR-expressing cells were collected by centrifugation (5,535 × *g* for 20 min), washed three times in 100 mM NaCl, and resuspended in the same solution for measurements. The cell suspension was then placed in the dark for several minutes and illuminated using a 300 W xenon lamp (ca. 14–16 mW/cm^2^, MAX-303, Asahi spectra, Japan) with a band-pass filter of 540 nm for 3 min by three times at an interval of 4 min. Measurements were repeated under the same conditions after the addition of the protonophore carbonyl cyanide m-chlorophenylhydrazone (CCCP) (final concentration, 40 μM). Light-induced pH changes were monitored using a Horiba F-72 pH meter and the light power was measured using an optical power meter (#3664, Hioki, Ueda, Japan) with an optical sensor (#9742, Hioki). All measurements were kept at 25 °C using a thermostat.

### HPLC measurements and pH titration experiments

The retinal composition was analyzed by HPLC using the purified RxR in a buffer containing 20 mM Tris-HCl (pH 7.0), 100 mM NaCl and 0.05 w/v % DDM. Before the measurements, the sample was stored at 4 °C for three weeks for dark adaptation and then extraction of retinal oxime from the sample was carried out with hexane after denaturation using methanol and hydroxylamine as described previously^14^. The sample was illuminated with green light (540 nm, ca. 7.0 mW/cm^2^) for 3 min to obtain the light-adapted RxR. The molar composition of each retinal isomer was calculated from the areas of the peaks in the HPLC patterns^18^. The pH titration experiments were performed using essentially the same method described previously^14^. In short, the experiments were performed at room temperature using purified RxR in a solution containing Good’s buffers, 0.89 mM citric acid, 0.89 mM MES, 1.1 mM TES, 0.78 mM TAPS, 1.1 mM CHES and 0.33 mM CAPS with 0.05% DDM. The buffer composition had the same buffer capacity over a wide range of pH values. The pH was then adjusted to the desired value by the addition of KOH or H_2_SO_4_. The sample was centrifuged at 20,631× *g* for 1 min (Model 3700 centrifuge with AF-2018 angle rotor, Kubota Corp., Tokyo, Japan) before the spectral measurement to remove aggregated proteins.

### Flash-photolysis and thermal stability

The flash-photolysis apparatus is equipped with a Xe flash lamp (PE-60SG, Panasonic Photo & Lighting Co., Ltd., Japan) as the actinic flash light source in combination with a yellow glass filter (Y-52, AGC, Japan). The experiments were performed using purified RxR in the buffer containing 50 mM Tris-HCl (pH 7.0), 1 M NaCl and 0.05% DDM at 60 °C.

To measure thermal stability, absorption spectral changes upon heat irradiation were monitored by a UV-Visible spectrometer as described previously^12^. To check the photoreactivity, we performed flash-photolysis measurements as well. During incubation, the suspension became turbid, maybe because of the aggregation of denatured proteins. Therefore, before the spectral measurement, the sample except for HsBR in PM was centrifuged at 20,631 × *g* for 2 min at 20 °C to remove any aggregates. The protein concentration of each sample was adjusted to 2 μM. The flash-photolysis experiments were performed using essentially the same method as described above. The experiments were performed using purified RxR, TR, and HsBR in DDM suspended in a buffer containing 50 mM Tris-HCl (pH 7.0), 1 M NaCl and 0.05% DDM, and HsBR in PM in a buffer containing 50 mM Tris-HCl (pH 7.0) and 1 M NaCl at 20 °C with Y52 filter.

## Additional Information

**How to cite this article:** Kanehara, K. *et al*. A phylogenetically distinctive and extremely heat stable light-driven proton pump from the eubacterium *Rubrobacter xylanophilus* DSM 9941^T^. *Sci. Rep.* **7**, 44427; doi: 10.1038/srep44427 (2017).

**Publisher's note:** Springer Nature remains neutral with regard to jurisdictional claims in published maps and institutional affiliations.

## Supplementary Material

Supplementary Figures
