# Supplementary material for: A phylogenetically distinctive and extremely heat stable light-driven proton pump from the eubacterium Rubrobacter xylanophilus DSM 9941T
Source: Sci Rep. 2017 Mar 14;7:44427. doi: 10.1038/srep44427 (PMC5349596; doi:10.1038/srep44427)
Supplement: Supplementary Figures [file srep44427-s1.docx]

**A phylogenetically distinctive and extremely heat stable light-driven proton pump from the eubacterium *Rubrobacter xylanophilus* DSM 9941^T^**

Kanae Kanehara^1^, Susumu Yoshizawa^2^, Takashi Tsukamoto^1, 3^ & Yuki Sudo^1, 3,^ *

^1^Division of Pharmaceutical Sciences, Okayama University, Okayama 700-8530, Japan.

^2^Atmosphere and Ocean Research Institute, The University of Tokyo, Chiba 277-8564 Japan.

^3^Graduate School of Medicine, Dentistry and Pharmaceutical Sciences, Okayama University, Okayama 700-8530, Japan.

*To whom correspondence should be addressed.

Yuki Sudo; Telephone: +81-86-251-7945; E-mail: [sudo@okayama-u.ac.jp](mailto:sudo@okayama-u.ac.jp)

# SUPPLEMENTARY INFORMATION


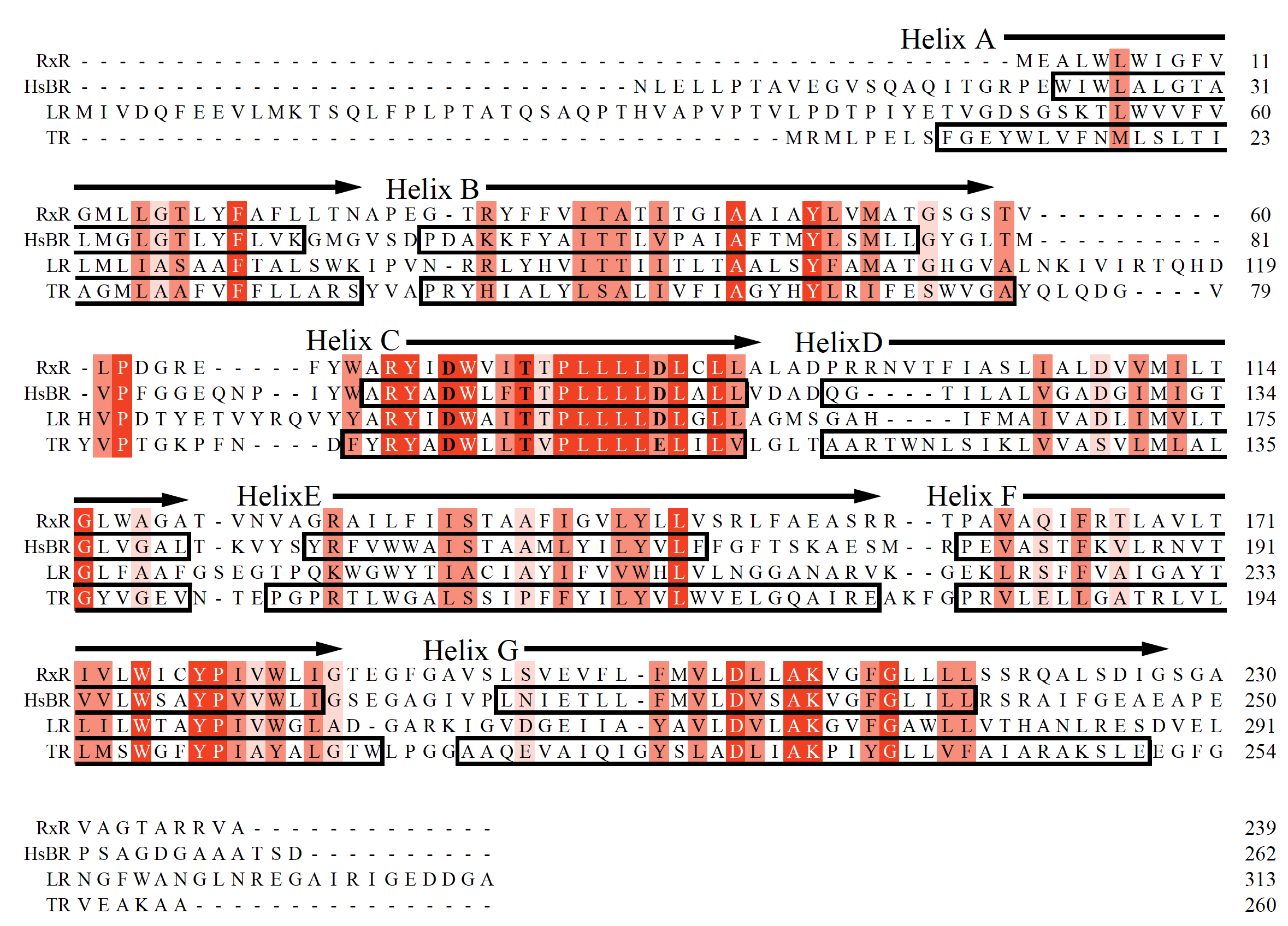


# Figure S1. Multiple sequence alignment of the proton pumping microbial rhodopsins

RxR, HsBR, LR and TR indicate *Rubrobacter xylanophilus* rhodopsin, *Halobacterium salinarum* bacteriorhodopsin, Leptosphaeria rhodopsin and thermophilic rhodopsin, respectively.

Helices A-G represent putative transmembrane regions.

**
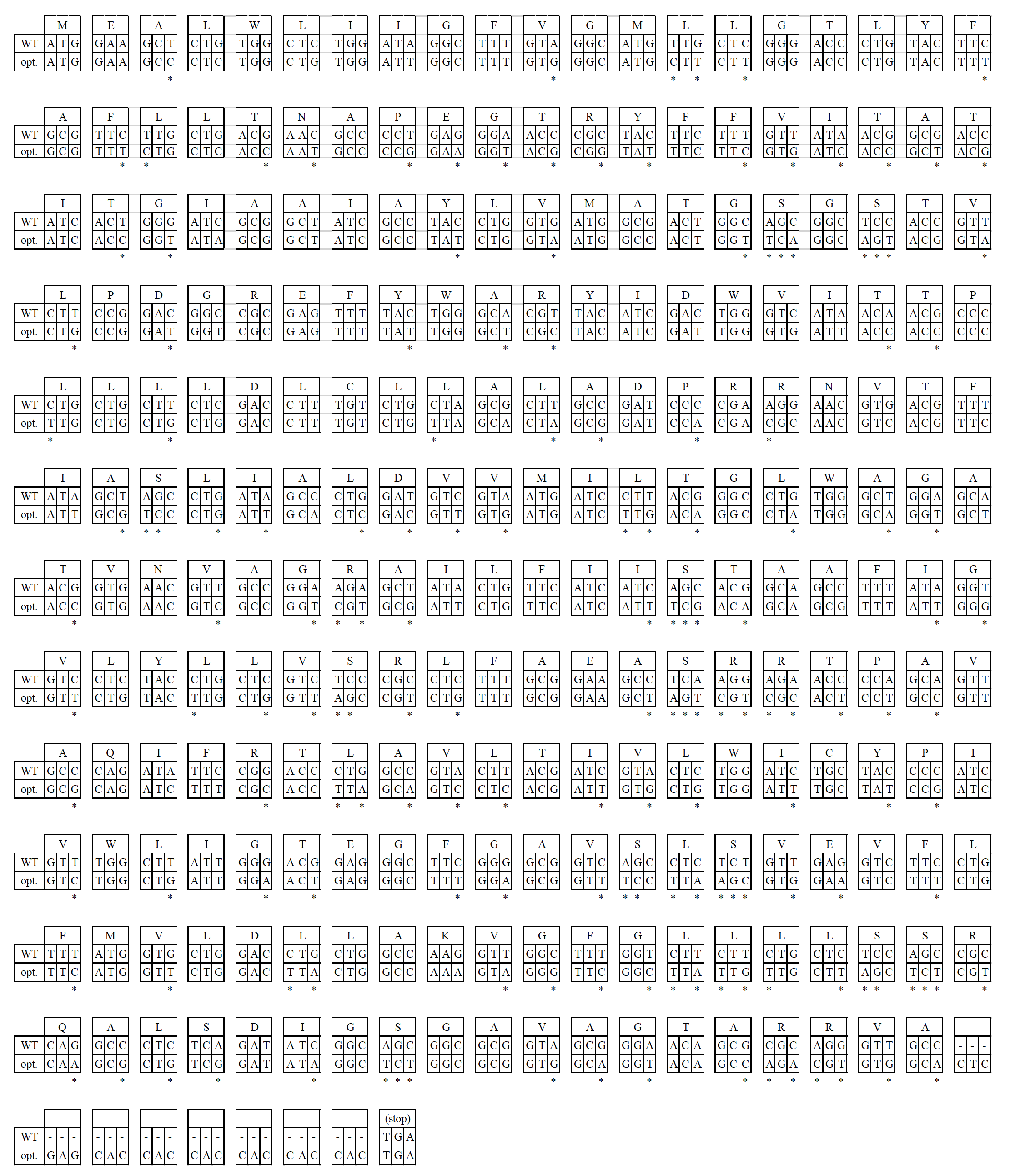
**

# Figure S2. Nucleotide sequence alignments of wild-type RxR (WT) and codon-optimized RxR for *E. coli* (opt.)

# Asterisks (*) show the optimized nucleotides for *E. coli*. During the optimization, some nucleotides (163 out of 717, 22.7%) were substituted but without any changes in the amino acid sequence.


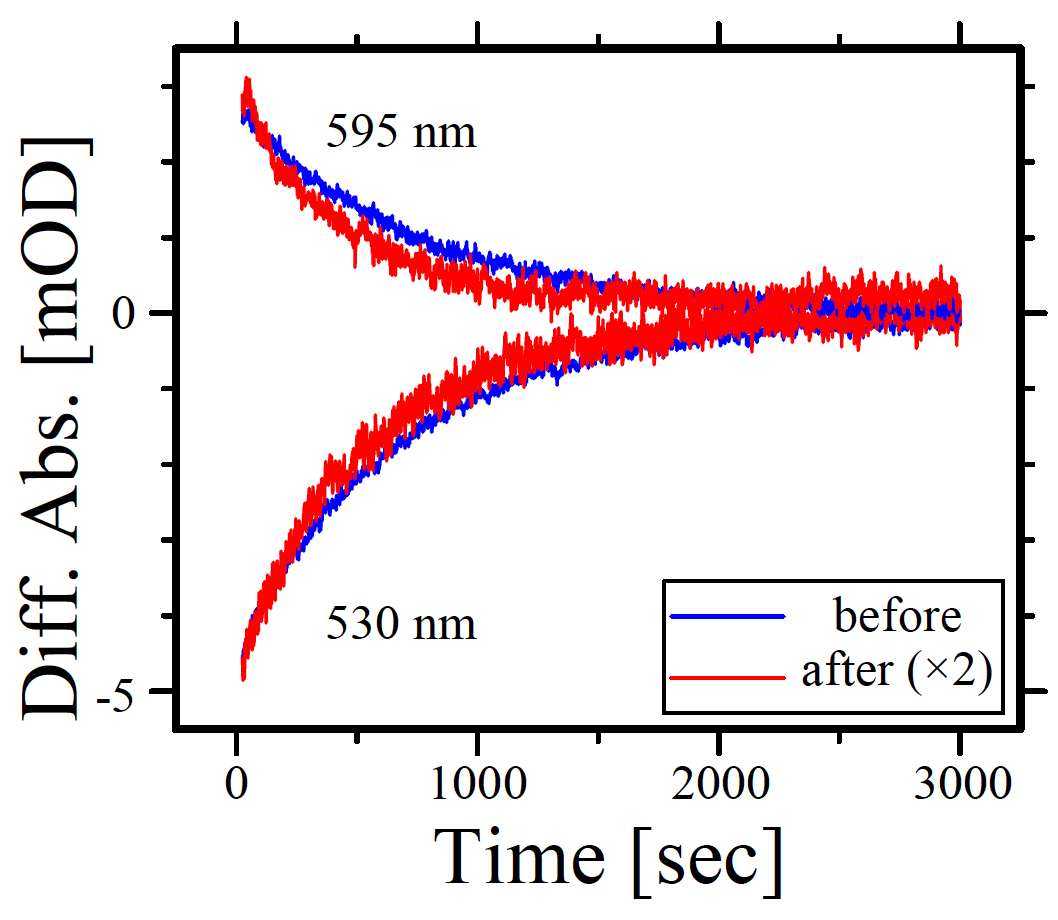


# Figure S3. The effect of heat irradiation on the photoreaction of RxR

# Flash-induced kinetic data of RxR before (blue) and after (red) heat irradiation for 600 min. The kinetics data after heat irradiation were scaled to be the similar intensity as to those before heat irradiation. The positive signals at 595 nm and the negative signals at 530 nm represent the absorbance changes of the RxR_O_ and the original state (RxR), respectively.
